# Fecal microbiota imbalance in Mexican children with type 1 diabetes

**DOI:** 10.1038/srep03814

**Published:** 2014-01-22

**Authors:** María Esther Mejía-León, Joseph F. Petrosino, Nadim Jose Ajami, María Gloria Domínguez-Bello, Ana María Calderón de la Barca

**Affiliations:** 1 Department of Nutrition and Metabolism, Centro de Investigación en Alimentación y Desarrollo, A.C., Hermosillo, Sonora, México; 2 Alkek Center for Metagenomics and Microbiome Research, Department of Molecular Virology and Microbiology, Baylor College of Medicine, Houston, Texas, USA; 3 Laboratory of Microbial Ecology, Department of Biology, University of Puerto Rico, San Juan, Puerto Rico; 4 Department of Medicine, New York University School of Medicine, New York, NY, USA

## Abstract

Dysbiosis of the intestinal microbiota affecting the gut barrier could be triggering Type 1 Diabetes (T1D), the second most frequent autoimmune disease in childhood. This study compared the structure of the fecal microbiota in 29 mestizo children aged 7–18 years, including 8 T1D at onset, 13 T1D after 2 years treatment, and 8 healthy controls. Clinical information was collected, predisposing haplotypes were determined; the fecal DNA was extracted, the V4 region of the 16S rRNA gene amplified and 454-pyrosequenced. The newly diagnosed T1D cases had high levels of the genus *Bacteroides* (p < 0.004), whereas the control group had a gut microbiota dominated by *Prevotella*. Children with T1D treated for ≥2 years had levels of *Bacteroides* and *Prevotella* compared to those of the control group. The gut microbiota of newly diagnosed T1D cases is altered, but whether it is involved in disease causation or is a consequence of host selection remains unclear.

Type 1 Diabetes (T1D) is a pro-inflammatory autoimmune disorder that results in β-cells destruction, leading to insulin deficiency^1^. T1D is one of the most frequent autoimmune disorders in childhood, with a worldwide prevalence of 1:200 to 1:300, which is higher than that reported a few decades ago^2^. The increasing incidence cannot be explained only by host genetic factors (HLA DR4-DQ8 and DR3-DQ2)^3^, and could instead reflect changes in early childhood exposure to risk factors^4^, such as lack of breastfeeding^5^ and viral infections^6^. Reported protection factors include microbial exposure^7^,^8^ and selective antibiotic treatments in early life^7^,^9^.

Associations between the intestinal microbiota alterations and B-cell autoimmunity have been described in HLA-DR/DQ genetically predisposed humans with positive GAD/IA2 autoantibodies^10^,^11^ or with T1D diagnosis^12^. Autoimmunity can be triggered after the translocation of microbes through the intestinal epithelium as a result of increased permeability^13^. Interestingly, the pancreas and gut endoderm share a common embryonic origin, which may create an immune link between both organs^14^.

The aim of this study was to evaluate the structure of the gut microbiota in genetically predisposed Mexican children with T1D at onset, and after treatment for more than 2 years.

## Results

### Study population

All subjects were Sonoran by birth, with at least 90% of the parents and grandparents born in northwest Mexico. A total of 21 T1D cases and 8 healthy controls were included. Eight cases were newly diagnosed T1D cases (less than 2 months evolution) and 13 were controlled with insulin treatment (with more than 2 years evolution [T1D treated for ≥2 years]) and HbA1C levels < 8%. Four subjects in the control group were siblings of T1D cases. The subjects' mean age for the 3 groups was statistically similar (Table S1).

### Clinical information

The number of antibiotic treatments per year, previous to diagnosis, was significantly higher (*p* = 0.043) in the T1D cases when compared with the antibiotic frequency in the same period in the controls (Table S1). The mean of antibiotic cycles per year in the newly diagnosed T1D cases was 4 cycles, while the T1D treated for more >2 year treatment group and controls had 6 and 3 cycles per year, respectively. At the time of the study, all subjects were on antibiotic-free scheme for at least 3 months prior to sampling. Other variables such as delivery mode, breastfeeding time, lactation regimes and infections per year, were not statistically different between controls and T1D cases (*p* ≥ 0.05).

### HLA types

All of the T1D cases (Table S1) had at least one HLA risk allele. The HLA DR3-DQ2 (DRB1*03/DQA1*0501/DQB1*0201) haplotype was in 19% of the cases, followed by 14.3% HLA DR4-DQ8 (DRB1*04/DQA1*0301/DQB1*0302/3) (Table S1). In addition, another 9.52% were heterozygous for the HLA DQ2-DQ8 haplotype. Furthermore, 75% of HLA DR3-DQ2 cases of T1D, were carriers of a DQ8 allele. Regarding DR alleles, 95% of the T1D children presented the DR4 variant (DRB1*04), while 66.6% were carriers of the DR3 allele (DRB1*03). In the control group, 12.5% were HLA DQ2. The rest of the control subjects were negative to DQ2 or DQ8; however, 37.5% presented isolated alleles associated with DQ8. All the control children were negatives for the DR alleles.

### Gut microbiota

**Figure 1:**
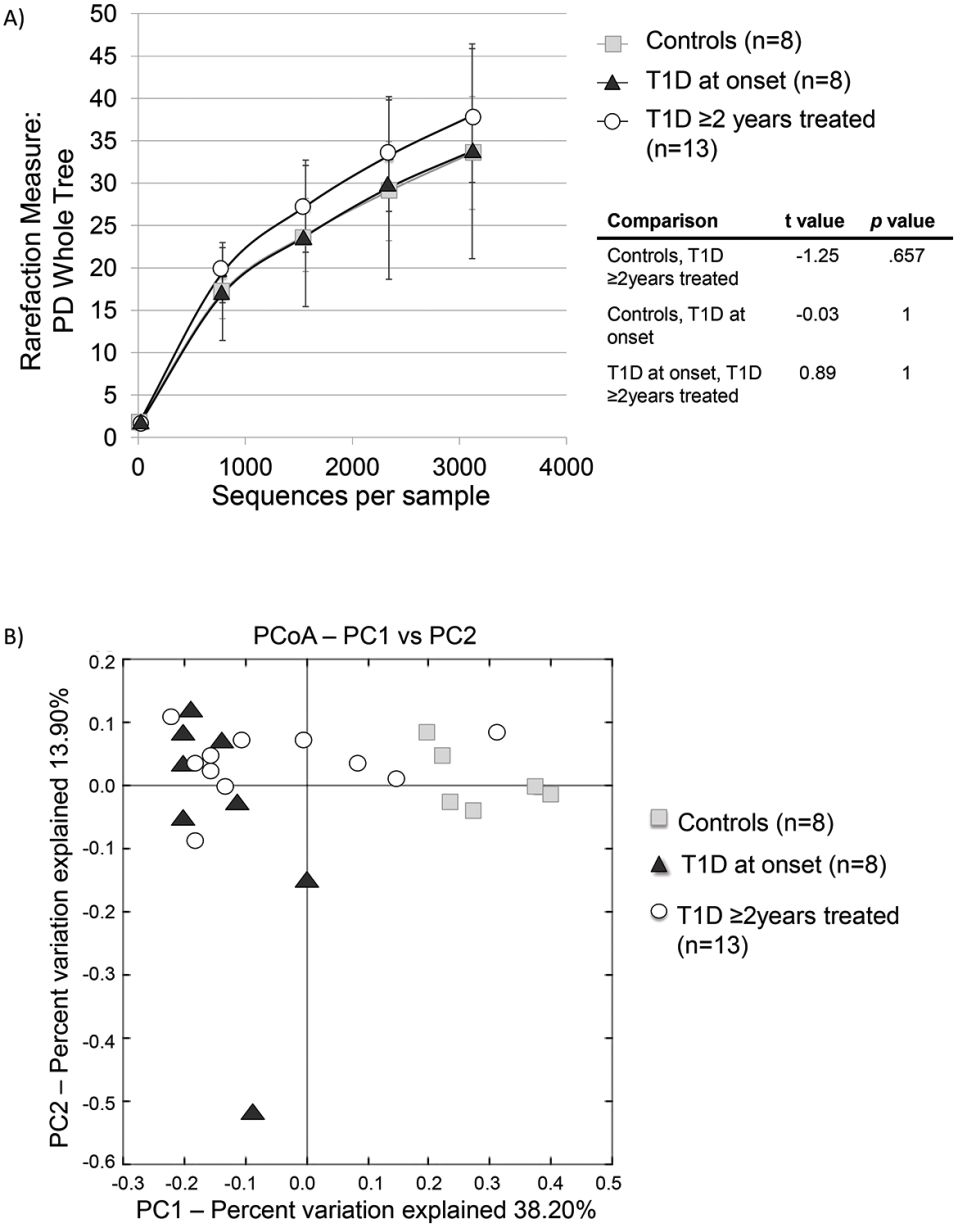
Diversity of the microbiota in T1D and healthy children. (A) Rarefaction curves of phylogenetic diversity in fecal samples (Student's t test); (B) Principal Coordinate Analysis of fecal communities weighted Unifrac distances in T1D and healthy children.

**Figure 2:**
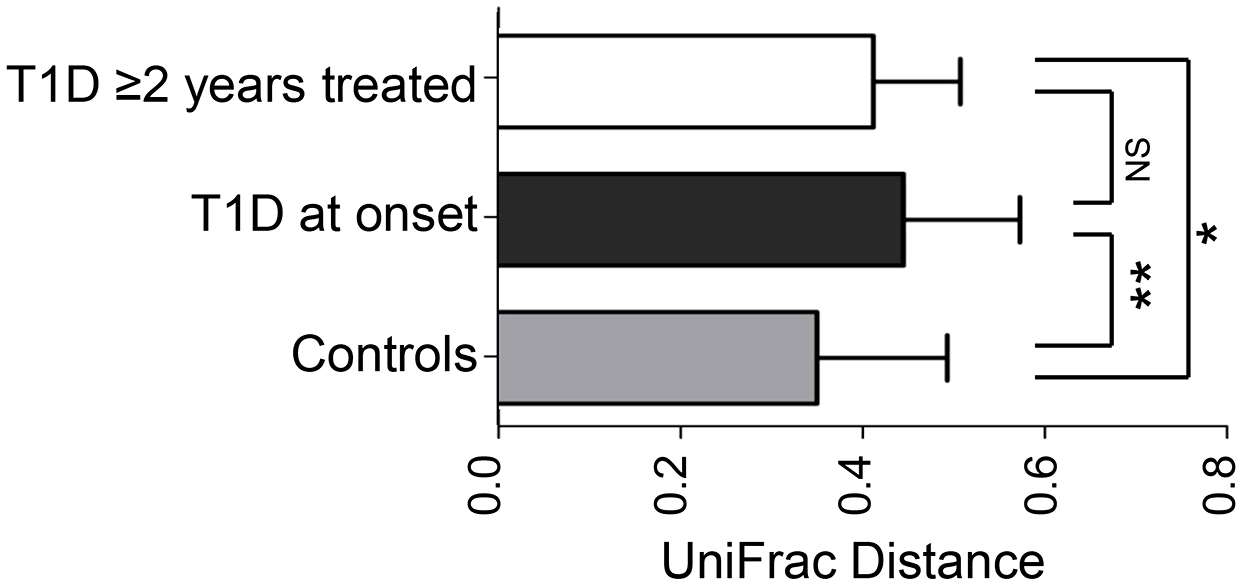
Weighted UniFrac distances between groups. *p,0.00005, **p,0.000005, NS: non-significant (Student's t test).

A total of 245,134 rRNA16S reads were obtained from the 29 samples, averaging 8,452 sequences per sample. The bacterial diversity was not significantly different between controls and the two T1D groups (Figures 1A, S1–2). However, beta diversity analysis using principal coordinates indicate a separate clustering of the fecal communities in subjects with T1D at onset compared to controls (Figure 1B). The group of T1D cases treated for 2 years did not show clustering and were spread along PC1 axis (Figures 1, 2, S3). Weighted UniFrac distances within groups were significantly smaller than between groups (*p* = 0.000001) thus indicating group clustering (Figure 2).

**Figure 3:**
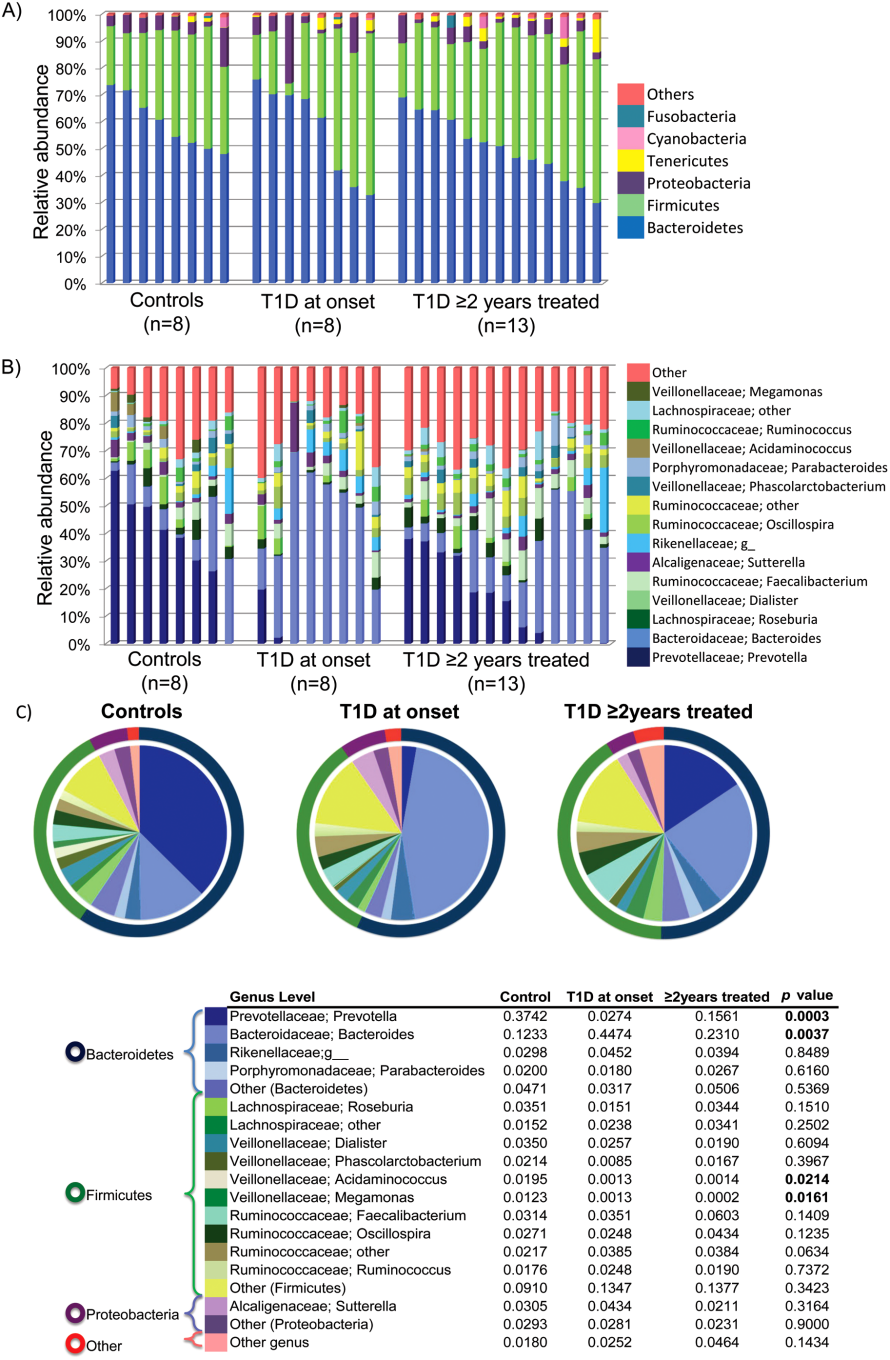
Average relative bacterial abundance of taxa in the fecal microbiota of healthy or T1D children. Relative abundance of fecal bacterial phyla (A) or genera (B) in each subject. Pie charts (C) depict phylum (outer ring) and genus (pie) level distribution. The proportions of the genus relative abundance are showed in the table and the *p* values of ANOVA, Tukey-Kramer test refer to the comparison between the 3 groups. In this analysis only phyla and genera with relative abundance greater than 1% were included. All OTUs with lower abundance were grouped as “other”.

The major taxa explaining this clustering were not reflected at the phyla but at the genus level (Figure 3). Control children had a fecal community dominated by *Prevotella*, while children with T1D at onset had higher representation of *Bacteroides* (*p* = 0.0037) showing substantially reduced proportions of *Prevotella* (*p* = 0.0003), *Megamonas* (*p* = 0.0161) and *Acidaminococcus* (*p* = 0.0214), in comparison to controls. Children with T1D treated for 2 years showed an intermediate relative abundance of these genera between the controls and T1D new cases (Figure 3, S4, S5). Dominant phyla were Bacteroidetes and Firmicutes, followed by Proteobacteria and Tenericutes, with no differences between groups (*p* > 0.05), and thus the ratio Bacteroidetes/Firmicutes was not significantly different between our groups.

## Discussion

T1D occurs typically in childhood or adolescence, and has been associated to host genetic factors with major histocompatibility complex (MHC) region harboring genes that contribute more than 50% of the risk, mediated by HLADR3-DQ2 or DR4-DQ8^15^.

Our results show that these in T1D Mexican children had higher proportions of HLA DR4 (95%) and DR3 (66.6%) than Mexican-American T1D children (46% and 27% respectively)^16^. In addition to genetic factors, environmental factors have also been epidemiologically associated to T1D. These include early life factors such as C-section^17^, lack of breastfeeding^5^ and early infections and antibiotic exposure^7^, all factors that are known to alter microbial communities^18^,^19^. The higher frequency of antibiotic treatments prior to diagnosis in T1D patients could influence the shift of the communities away from the controls; however, T1D at onset group had received fewer courses than the group of T1D treated for 2 years, and yet, had the most distinct community.

There was higher dispersion in the structure of the gut microbiota in T1D cases, than in controls (Figure 2). This finding is consistent with those found in previous studies related to autoimmunity and T1D^10^,^12^, suggesting instability of the microbiome associated with this condition.

The fecal microbiota in Mexican healthy children of our study had a similar composition to that reported in people from developing countries with high levels of *Prevotella*, in contrast with the *Bacteroides*-dominated microbiota of US children and adults^20^,^21^. These differences are likely to be dietary, since *Bacteroides* has been associated with high protein and fat diets, while carbohydrate rich diets, increase *Prevotella*^22^. It is not clear if high dominance of *Bacteroides* in the US is associated with higher risk of TD1, which is indeed higher in US than in Mexican children (11 to 17 vs 1.5 per 100,000, respectively)^23^. The incidence of T1D in the Sonora region -near the US-Mexican border- has doubled in the last 10 years^24^, suggesting that the factors underlying risks are environmental, and could be related to diet or lifestyle changes.

*Bacteroides*-dominant gut communities were also observed in prediabetic Finish children who also showed decreased levels of *Prevotella* when compared to healthy controls^25^. Insulin treatment of T1D partially normalizes the microbiota profile towards *Prevotella*-dominant profile, indicating that physiological changes related to T1D are driving the gut microbiota structure in T1D. It has been suggested that *Bacteroides* activity in mucine-synthesis and degradation might be contributing to T1D development by thinning of the mucus layer, leading to increased gut permeability and inflammation^26^. Lactate conversion to butyrate in the gut induces synthesis of mucins and consequently favors a healthy epithelium. *Bacteroides* would transform lactate into short chain fatty acids, such as propionate, acetate and succinate, which contribute to decrease mucin synthesis and therefore the tight junctions could be affected^25^. Thus, the lactate model seems to be critical to maintain intestinal health and explain the way to autoimmunity in T1D.

Bacterial antigens and microorganisms toxins can be sensed by molecules related to epithelial cells' tight junctions like zonulin, claudin and occludin, altering their activity and consequently increasing gut permeability and bacterial translocation^27^. In this context, some *Bacteroides sp.* with pathogen activity, such as *Bacteroides fragilis*, disrupts the tight junctions by proteolytic degradation due to metalloprotease enterotoxins (e.g. fragilysin) increasing paracellular permeability with local inflammation, cell damage and loss of microvilli^28^. This process could lead to loss of self-tolerance, causing aberrant immune responses, with an homeostatic imbalance of T-cells, gut inflammation and extra intestinal inflammatory infiltrate «insulitis» associated with T1D^29^.

The microbiota profile associated to T1D supports the idea of the existence of an “autoimmune” microbiome associated with diabetes^10^. However, this association does not necessarily imply causality. Intervention studies where the gut microbiota structure could be normalized in children at high risk for T1D via fecal transplant, have not yet been performed, but it could demonstrate an etiologic role of dysbiosis in T1D.

## Methods

### Study population

A cross-sectional case-control study was conducted in the Mexican Sonora state, between 2010 and 2012, recruiting children T1D patients at the Children's Hospital of the State of Sonora (HIES). The criteria for inclusion comprised being of 7 to 18 years of age, a T1D diagnose as established according to the American Diabetes Association criteria^1^, a positive anti-GAD and/or anti-IA-2 auto-antibodies result, HbA1C levels < 8%, and an antibiotic-free scheme for at least 3 months prior to the sampling. Subjects presenting chronic and inflammatory gastrointestinal diseases were excluded from the study.

In T1D patients, the insulin dose is currently carefully calculated based on the carbohydrate content of the diet and the physical activity. In general, a high-fiber diet with low glycemic index is recommended^30^. All patients' parents were informed about the nature of the study and were asked to provide written informed consent for their child's participation. All patients had nutritional counseling.

Information about birth delivery mode, breastfeeding, complementary feeding patterns in the first year of life, infections and antibiotic treatments prior to T1D diagnosis was collected in an interview with the parent. Clinical records were revised, and the protocol was approved by the Ethics Committee of the Centre for Food Research and Development and the HIES Learning and Research Board.

### Sample collection

Peripheral blood (2 mL) was collected from all patients and stored in EDTA tubes at 4°C for haplotype analysis. Stool samples were obtained from every subject and immediately placed on ice for transportation to the laboratory. The DNA extraction was performed within the first 3 h after collection.

### gDNA extraction

Genomic DNA was extracted from whole blood samples using the QIAamp DNA Blood Mini Kit (QIAGEN®), following manufacturer's instructions. For stool samples the QIAamp DNA Stool Mini Kit (QIAGEN®) was used. The concentration and purity were evaluated using a Nanodrop ® spectrophotometer (Thermo Scientific, Wilmington, DE, USA). Extracted DNA was stored at −20°C.

### HLA typing

The HLA DR3-DQ2 and DR4-DQ8 typing was performed from blood gDNA through polymerase chain reaction (PCR) using sequence specific primers^31^ for DRB1*03, DRB1*04, DQA1*0501, DQA1*0301, DQB1*0201 and DQB1*0302/3 alleles (IDT-Integrated DNA Technologies, Tucson-AZ, USA). The amplicons obtained were resolved on 1.8% agarose gel, stained with GelRed™ (Biotium Inc., Hayward, CA, USA) and visualized under ultraviolet light (Molecular Imager®Bio-Rad, Hercules, CA, USA). The allele HLA frequencies in both clinical groups were calculated using the direct counting method.

### Microbiota determination

The V4 region of the bacterial 16S rRNA gene was amplified from fecal gDNA with 357F/926R primers containing the A and B adaptors from 454 Life Sciences for pyrosequencing, and a unique 12 base pair error correcting Golay barcode, allowing multiplexing of samples in one single run. Replicate amplicons were pooled and purified using the UltraClean –htp 96 well PCR clean up kit (MO BIO). The concentration of samples was measured and samples were combined in equimolar ratios into a single tube. Pyrosequencing was carried out on a 454 Life Sciences Genome Sequencer FLX instrument (Roche) by the Human Genome Sequencing Center at Baylor College of Medicine, in Houston, Texas, USA, using their standard protocol.

### Bioinformatics and community comparisons

The sequence and microbial communities diversity analysis was performed with the QIIME software package (Quantitative Insights Into Microbial Ecology)^32^. Sequences were removed from the analysis if they were <200 nt, had a quality score < 25, contained ambiguous characters, contained an uncorrectable barcode, or did not contain the primer sequence. Sequences were assigned to samples by examining their individual 12-nt barcode. Sequences were aligned using PyNAST, clustered into OTUs and taxonomy assigned using the Ribosomal Database Project (RDP) as a reference base. The phylogenetic tree was built with FastTree. QIIME and UniFrac were used for analyses of bacterial communities and group comparisons^33^. Differences between groups were assessed using ANOVA and Tukey-Kramer multiple-comparison test.

## Author Contributions

M.E.M. and A.M.C. designed and conducted the study, collected samples, analyzed haplotypes, extracted DNA, analyzed data and wrote the main manuscript text, J.F.P. and N.J.A. run the microbiota pyrosequence and suggested data analyses methods and M.D.B. supervised the analysis data and wrote the main manuscript text. All authors reviewed the manuscript.

## Supplementary Material

Supplementary Information Supplementary Information
