# Supplementary material for: Fecal microbiota imbalance in Mexican children with type 1 diabetes
Source: Sci Rep. 2014 Jan 22;4:3814. doi: 10.1038/srep03814 (PMC3898044; doi:10.1038/srep03814)
Supplement: Supplementary Information [file srep03814-s1.pdf]

Fecal microbiota imbalance in Mexican children with type 1 diabetes

María Esther Mejía-León, Joseph F Petrosino, Nadim Jose Ajami, María Gloria Domínguez-Bello and Ana María Calderón de la Barca

Table S1 | Sample characteristics

	T1D cases	Controls	*p* value
Age in years (SE)			0.3
	12.3 (0.64)	11 (1.04)	
Delivery mode			0.092
Cesarean	6	5	
Vaginal	15	3	
Breastfeeding time			0.185
<6 months	11	2	
≥6 months	10	6	
Milk formula start			0.154
<4months	14	3	
≥4 months	7	5	
Infections per year			0.092
≥5	15	3	
<5	6	5	
Antibiotic treatments per year			0.043
≥5	14	2	
<5	7	6	
HLA types « subjects (%) »			
Presence of any risk allele	21 (100)	4 (50)	
DR3-DQ2	4 (19)	1 (12.5)	
DR4-DQ8	3 (14.3)	0 (0)	
DQ2-DQ8	2 (9.52)	0 (0)	
3 isolated risk alleles	7 (33.3)	0 (0)	
2 isolated risk alleles	3 (14.3)	0 (0)	
1 isolated risk alleles	2 (9.52)	3 (37.5)	
Negative	0 (0)	4 (50)	

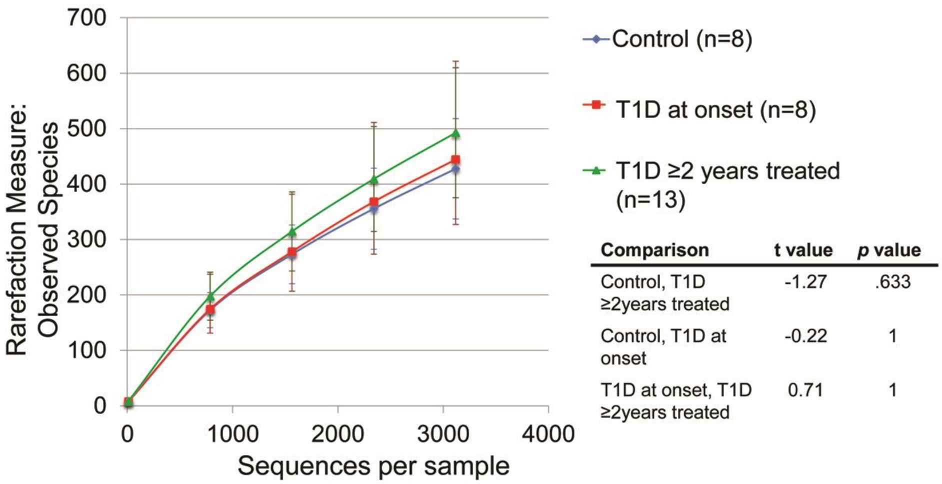

Comparison	t value	*p* value
Control, T1D ≥2years treated	-1.27	.633
Control, T1D at onset	-0.22	1
T1D at onset, T1D ≥2years treated	0.71	1

Figure S1│Rarefaction curves of observed species in fecal samples from T1D children between 7-18 years old and healthy controls.

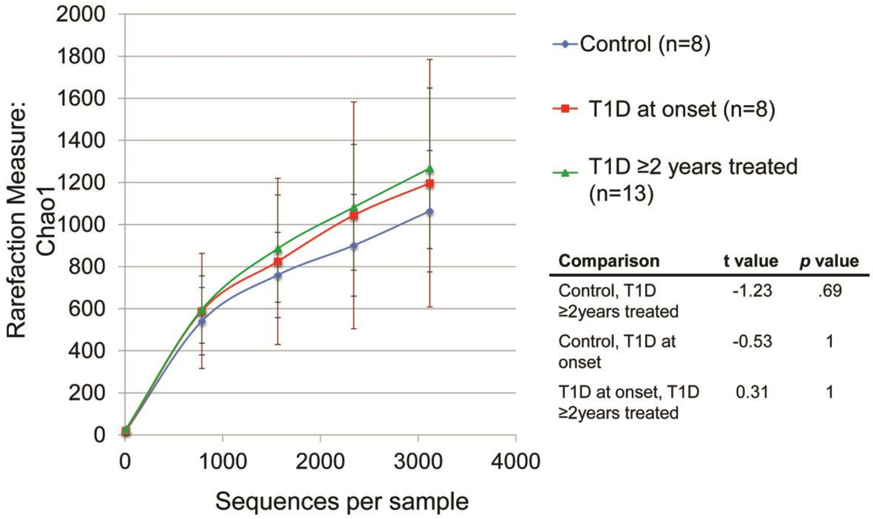

Comparison	t value	*p* value
Control, T1D ≥2years treated	-1.23	.69
Control, T1D at onset	-0.53	1
T1D at onset, T1D ≥2years treated	0.31	1

Figure S2│Rarefaction curves of Chao1 in fecal samples from T1D children between 7-18 years old and healthy controls. There were no significant differences between control and T1D groups.

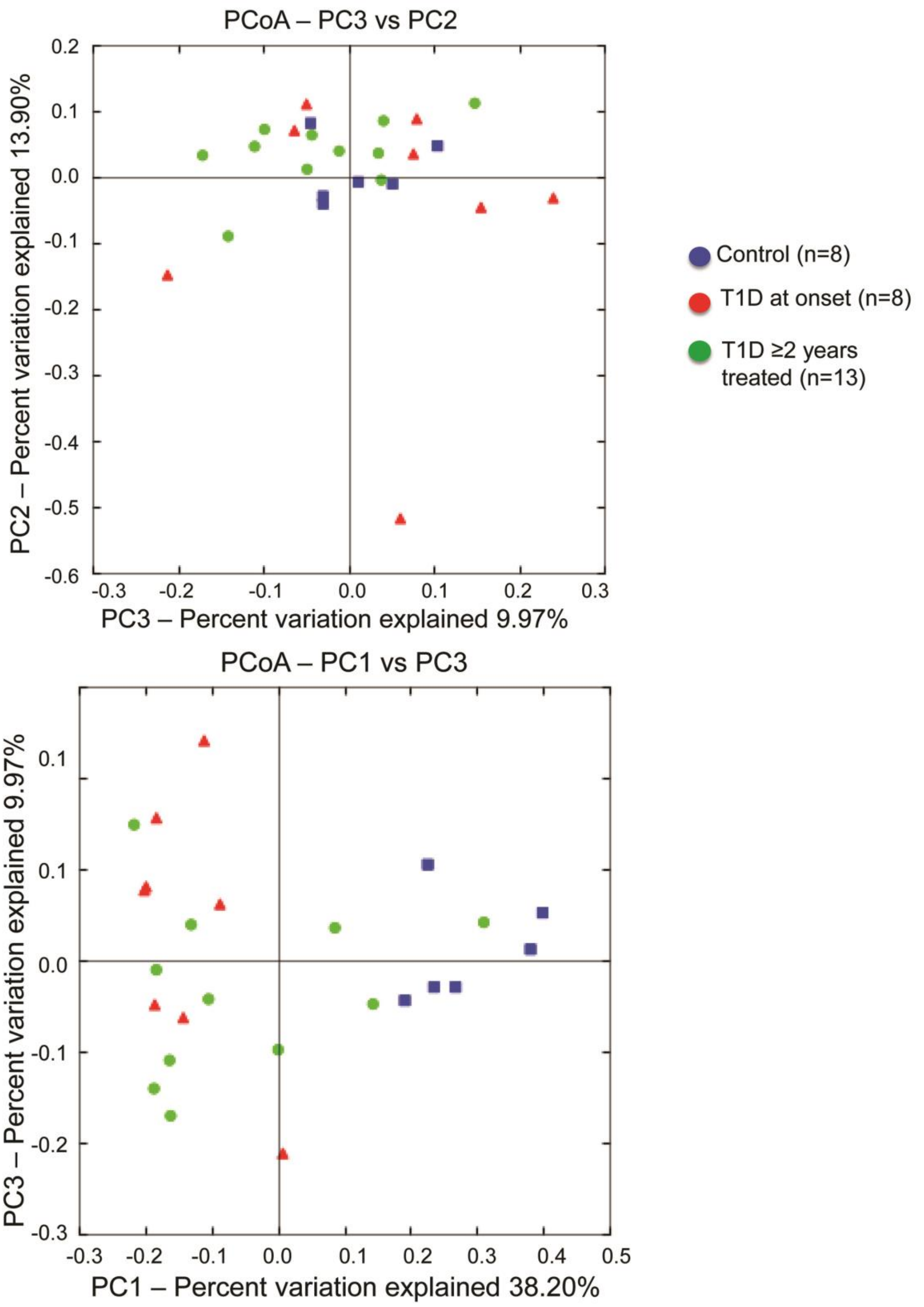

Figure S3│ Principal Coordinate Analysis of fecal communities Unifrac distances in T1D and healthy children (components 2 and 3; weighted analysis). Principal coordinate 1 separates controls from T1D onset with the T1D>2y distributed between the two groups.

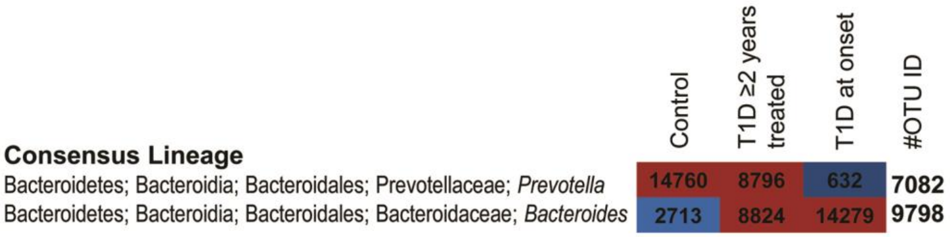

Figure S4│Heat map of the relative abundance Prevotella and Bacteroides in each children group. Controls have high dominance of Prevotella, and T1D onset has high dominance of Bacteroides, with the T1D>2y distributed between the two types.

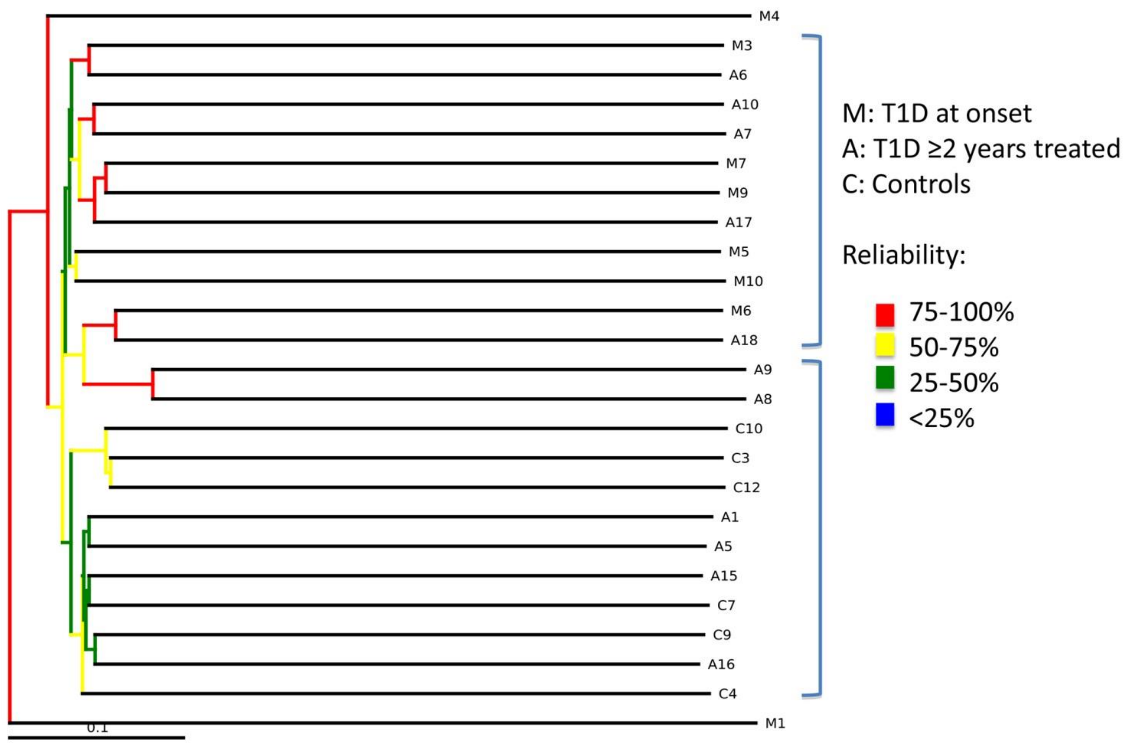

Figure S5│Clustering of fecal communities in T1D groups and controls based on phylogenetic distances. M (T1D onset) clusters in upper cluster and controls cluster in the bottom cluster with A (T1D >2y), distributed in the two clusters.
